# Choosing Actions

**DOI:** 10.3389/fpsyg.2013.00273

**Published:** 2013-06-03

**Authors:** David A. Rosenbaum, Kate M. Chapman, Chase J. Coelho, Lanyun Gong, Breanna E. Studenka

**Affiliations:** ^1^Department of Psychology, Pennsylvania State University, University Park, PA, USA; ^2^Department of Health, Physical Education, and Recreation, Utah State University, Logan, UT, USA

**Keywords:** action selection, degrees-of-freedom problem, motor control, behavioral psychology, choosing actions

## Abstract

Actions that are chosen have properties that distinguish them from actions that are not. Of the nearly infinite possible actions that can achieve any given task, many of the unchosen actions are irrelevant, incorrect, or inappropriate. Others are relevant, correct, or appropriate but are disfavored for other reasons. Our research focuses on the question of what distinguishes actions that are chosen from actions that are possible but are not. We review studies that use simple preference methods to identify factors that contribute to action choices, especially for object-manipulation tasks. We can determine which factors are especially important through simple behavioral experiments.

## Introduction

Actions make psychological activity tangible, for it is through actions that decisions are expressed. To be on the frontier of psychology, therefore, it is desirable not just to know what actions are chosen but also how they are. The actions of interest can be large-scale, as in deciding whether to stay in school or drop out; or they can be small-scale, as in raising one’s eyebrow or nodding in a way that conveys less than full agreement. The actions need not be communicative, however. They can be purely functional, as in reaching for a cup of coffee when one is alone. Such functional actions can also be carried out in different ways, quickly and assuredly, for example, or slowly and hesitantly.

Psychologists have paid little attention to the way actions are physically expressed. Instead, they have typically focused on the instrumental outcomes of behavior, the most famous example being B. F. Skinner’s research, in which rats pressed on levers or pigeons pecked on keys to get rewards or avoid punishments (e.g., Skinner, 1969). How the rats pressed the levers or how the pigeons pecked the keys were of less interest than which devices were activated when.

The restriction of focus to switch closures, whether achieved with limbs or beaks, is understandable when one’s methods of recording behavior are primitive. It is much easier to record which electrical switch is closed in a Skinner box than to quantify the detailed properties of movement trajectories. Still, the manner in which movements are made may be relevant not just for conveying subtleties of communication or for determining whether a task is performed confidently. How movements are made may also be relevant for shedding light on motor control itself.

Consider the simple act of pressing an elevator button. An elevator summoned by a button press is indifferent to the movements made to press the button. Still, the movements made to press the button are a concern for the person pressing the button. This is obvious for someone with a movement disability, but even for neurologically typical individuals, there is a non-trivial problem to be solved in pressing an elevator button. The number of possible joint configurations that let the finger press the button is limitless. In addition, for any given joint configuration achieved at the time of the press, the number of paths leading to that joint configuration is limitless as well. Finally, for every one of those paths to the final configuration, the timing possibilities are boundless, too. So even for a task as trivial as pressing an elevator button, the number of possible actions is infinite. A core question in motor control is how, for situations like this, particular actions are chosen.

## Approaches to Action Selection

The problem of choosing actions in the sense just discussed was first recognized by Bernstein (1967), who referred to the matter as the *degrees-of-freedom* problem. As Bernstein appreciated, the degrees of freedom of the body exceed the degrees of freedom associated with the ostensive description of most tasks to be achieved. An elevator button, for example, has six (positional) degrees of freedom – the three spatial coordinates of its center, and the three orientation coordinates of its plane (pitch, roll, and yaw). The width of the button (governing its tolerance for aiming errors) is relevant as well, as is the force needed to complete the press. Summing up these degrees of freedom, there are eight of them.

The degrees of freedom of the body of a typical person intent on pressing a button are vastly greater. Considering only the skeleton, a person’s upper arm has three degrees of freedom (rotation about the *x*, *y*, and *z* axes), the forearm has two degrees of freedom (flexion/extension and twisting), and each finger joint adds its own degrees of freedom. Adding the joints of the spine, hip, knee, and ankle, still more degrees of freedom come along. How the head is oriented enters as well, how the eyes are oriented factors in, and so on. Quickly, the bodily degrees of freedom exceed the eight associated with the button, and this ignores the vicissitudes of the muscles affecting the joints and the nerves driving the muscles, which create an even greater explosion of possibilities.

### Coupling

How can one make progress on the challenge of choosing particular actions when infinitely many achieve a task? In the literature on this topic three approaches have been taken. Two were pursued by Bernstein (1967). A third emerged after him.

One approach that Bernstein (1967) pursued was to identify functional dependencies between effectors. Bernstein’s idea was that linkages between effectors could limit the degrees of freedom to be controlled.

**Figure 1:**
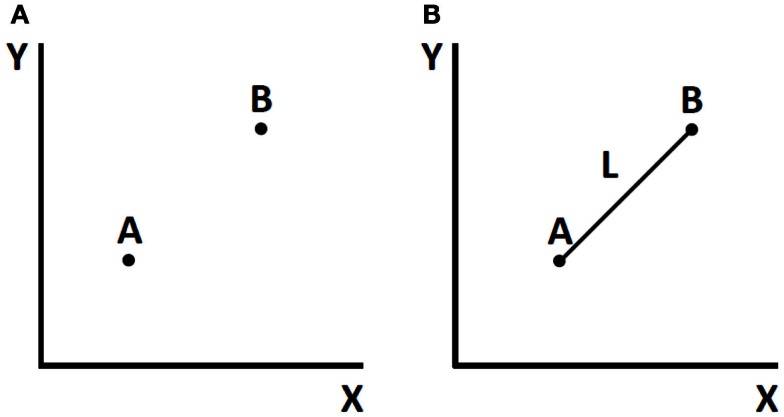
**Effects of coupling on degrees of freedom**. **(A)** Two independent points (four degrees of freedom). **(B)** Two points joined by a line of immutable length (three degrees of freedom).

At an abstract level, this approach can be appreciated by considering Figure 1, which shows, in one case, two independent points in a plane and, in the other, two points joined by a line of fixed length. In the first case, there are four degrees of freedom: the *x* and *y* values of point A, and the *x* and *y* values of point B. In the second case, there are three degrees of freedom: the *x* and *y* of one point and the angle of the line, whose length is fixed, from A to B. This simple example, adapted from Saltzman (1979), shows how coupling can reduce the degrees of freedom to be managed.

Does coupling exist in actual motor performance? The answer, resoundingly, is Yes. As noticed by von Holst (1939), when fish oscillate their dorsal fins and then start to oscillate their pectoral fins, the dorsal fin oscillations change. When von Holst asked human subjects to do something similar, raise and lower one outstretched arm at a fixed frequency and then at other frequencies, the oscillations of the control arm changed. Such limb interactions occur reliably and have been studied in detail (e.g., Swinnen et al., 1994).

What do these results imply about the degrees-of-freedom problem? They might be taken to suggest that dependencies between effectors obviate the problem, but there is a difficulty with this suggestion. Linkages are not fixed but rather come and go depending on what needs to be achieved. During speech, for example, the upper lip moves down toward the lower lip more quickly than usual if the lower lip rises more slowly than usual (and vice versa), but this is only true when the sound to be produced requires bilabial closure, as in “p” or “b.” It is not the case when the sound to be produced is a fricative, as in “f” or “v” (Abbs, 1986).

The manifestation of coupling also depends on how the task is presented. When the perceptual representation of the task is simplified, actions that are otherwise difficult to perform can be easy (Mechsner et al., 2001). Similarly, if the hands haptically track moving objects, staying in light touch with the objects while the objects move, two circularly moving objects turning at different frequencies can be haptically tracked essentially perfectly no matter what the frequency relation between them. By contrast, generating two circles with those same frequencies is nearly impossible if the circles are drawn through more conventional means, such as drawing them on a blackboard (Rosenbaum et al., 2006b).

### Mechanics

The second track that Bernstein (1967) pursued to address the degrees-of-freedom problem was to appeal to exploitation of mechanics. His idea was that action control can be simplified by exploiting mechanical interactions between the body and outer world.

Examples of motor performance that reflect exploitation of mechanics abound. A delightful example concerns babies in Jolly Jumpers. Suspended in their little seats, dangling via elastic chords from firm hooks above, babies learn to push on the floor at just the right pace and force to get the most “bang for the buck” (Goldfield et al., 1993).

Once babies and toddlers learn to walk, they continue to exploit mechanics. During mature walking there is a stance phase and a swing phase for each foot. During the stance phase the foot is *on* the ground, whereas during the swing phase the foot is *off* the ground. During the swing phase there is remarkably little muscle activity once the swing is initiated. The swing is completed, however, because the leg is swung forward and then pulled down via gravity. It turns out that people switch from walking to running as locomotion speed increases at just the speed where leg lowering would occur more quickly than is achievable by letting gravity pulling the leg down. At this critical speed, the transition is made from walking (a series of controlled falls), to running (a series of controlled leaps) (Alexander, 1984).

Does exploitation of mechanics solve the degrees-of-freedom problem? Perhaps to some extent in some circumstances. For example, exploitation of mechanics has been shown to be a useful way to avoid copious computation for robot trajectories (Collins et al., 2005). Still, it is unclear how far one can go with this approach, for it fails to explain the richness and diversity of voluntarily shaped performance.

### Constraints

If neither the coupling approach to the degrees-of-freedom problem nor the mechanics approach to the degrees-of-freedom problem fully solves the problem, what approach can do so? Toward answering this question, it is useful to return to the way we introduced the degrees-of-freedom problem earlier in this article. We noted that Bernstein (1967) couched the problem in terms of the degrees of freedom of the body relative to the degrees of freedom associated with the ostensive description of the task to be achieved. The key phrase for us as psychologists is “ostensive description.” What we mean is that while a task description has *some* properties, the individual approaching the task adds more properties to the description – enough of them, in fact, to fully describe the problem and thereby, in effect, solve it. For example, if the task is “to press the elevator button,” the person about to perform this task might add more constraints, such as “… with an effector that can easily be brought to the button.” The effector might be the right index finger, but if the individual were holding a squirming baby, some other effector might be used instead.

Saying that constraints limit action choices raises the question of how scientists can identify those constraints. To begin with, note that if constraints limit the range of possible actions, the constraints that do so correspond to the features of actions that are performed. Similarly, actions that could achieve the task but are not performed lack those features. Not all constraints are equally important, however. If an elevator button must be pushed, it is probably more important to press the button with a finger than to carry one’s finger to the button with some desired average speed.

Given this pair of points – that constraints are mirrored in the features of selected actions and that some constraints are more important than others – the challenge for psychologists interested in action selection is to discover which constraints are more important than which others. Determining the ranking or weighting of constraints achieves two things. First, it obviates the need to say which constraints are relevant and which are not. That is, instead of adopting such a binary classification, all possible constraints can be, and indeed must be, included. What distinguishes the constraints, then, is their weights. Some constraints have large weights. Others have small weights, including weights that are vanishingly small (i.e., nearly zero or zero itself).

Second, the weights of the constraints define the task as represented by the actor. This point is of inestimable importance for psychology because so much of psychological research is about performance of one task or another – the Stroop task, the Flanker task, and so on. What a task is – how it is represented by someone performing it – is rarely considered, but the issue is core to understanding action selection and psychology more broadly. If a bus driver sees his task as setting people straight about how to enter his bus, then the way his passengers feel about him will be very different than if he sees his task as greeting his passengers as warmly as he can.

**Figure 2:**
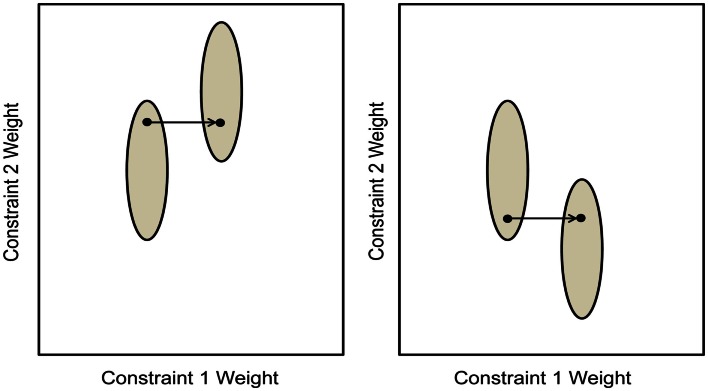
**Actions selected from those that satisfy elementary task demands (enclosed in ellipses) defined by their locations in the two-dimensional space of weights (0–1) for constraint 1 and constraint 2**. A different first point is chosen in the left case and second.

A mathematical formalism can help pave the way for where we will go with this. The formalism lets us depict tasks in an abstract “task space” (Figure 2) and lets us introduce a hypothesis about minimization of transitions within this space.

An elementary task, T, performed at time 1 can be defined as a vector of constraint weights, *w*_1,1_ for constraint 1, *w*_2,1_ for constraint 2, and so on, all the way up to constraint *n* at time 1.

$$T=\left[\;\begin{array}{c} w_{1,1}\\ w_{2,1}\\ \cdot \\ \cdot \\ \cdot \\ w_{n,1} \end{array}\right]$$

The weights can be visualized as a point in task space, as seen in Figure 2. The axes of the space correspond to the weights (between 0 and 1) for the possible constraints. Figure 2 shows just two constraint weights, for graphical convenience. In this illustration, ellipses contain the possible weights for achieving a given elementary task. A single point within the ellipse is highlighted to show which weight combination is chosen.

It is also possible to consider *series* of elementary task solutions, as shown in the next equation, where we extend the first equation to one in which all the weights take on values for a range of times from time 1 up to time *t*:

$$T=\left[\;\begin{array}{ccccc} w_{1,1} & w_{1,2} & w_{1,3} & \ldots & w_{1,t}\\ w_{2,1} & w_{2,2} & w_{2,3} & \ldots & w_{2,t}\\ \cdot & \cdot & \cdot & \cdot & \cdot \\ \cdot & \cdot & \cdot & \cdot & \cdot \\ \cdot & \cdot & \cdot & \cdot & \cdot \\ w_{n,1} & w_{n,2} & w_{n,3} & \ldots & w_{n,t} \end{array}\right]$$

Two task series are shown in Figure 2. In the case on the left, the first elementary task solution takes into account which point will be chosen for the second elementary task. In the right panel, though the set of possible solutions for the first elementary task is the same as in the left panel, the weighting pair chosen within it is different. The reason is that a different task is required next.

What we are saying is that actions may be selected in a way that minimizes transitions through task space. This idea has been appreciated before (e.g., Jordan and Rosenbaum, 1989) and is particularly well known in connection with speech co-articulation, where the way a sound is produced depends on what sounds will follow (Fowler, 2007).

## Object Manipulation

In our laboratories at Penn State and Utah State, we have been concerned with manual control rather than speech control. Our particular interest within the domain of manual control has been object manipulation. Object manipulation is particularly interesting to us because we take a cognitive approach to action selection. In studies of object manipulation the same object can be used for different purposes. A pen can be used for writing or for poking, a knife can be used for slicing or for jabbing, and so on (Klatzky and Lederman, 1987). This feature of object manipulation makes the associated tasks attractive to us given our cognitive bent. The same participant can be exposed to the same object in the same position and can be instructed or otherwise induced to use the object with different goals. Differences in the way participants grasp or handle the object depending on the future task demands can be ascribed to differences in the participants’ mental states.

### Order of planning

Yet another attraction of object manipulation is that one can study planning effects of different orders. One can look for *first-order* planning effects, reflecting the influence of the object being reached for in its present state; or one can look for *second-order* planning effects, reflecting the influences of what is to be done *next* with the object; or one can look for *third-order* planning effects, reflecting the influences of what is to be done *after that*; and so on (Rosenbaum et al., 2012). The highest-order planning effect that can be observed can be taken to reflect the planning span. For discussions of planning spans for speaking and typewriting, see Sternberg et al. (1978) and Logan (1983).

As long as there are second- or higher-order planning effects in object manipulation, those effects can be viewed as manual analogs of speech co-articulation. We can, in fact, coin a phrase to highlight this association. Just as there are co-articulation effects for speech, we can say there might be “co-manipulation” effects for manual control.

One would expect co-manipulation effects if the cognitive substrates of co-articulation extended to manual behavior. Saying this another way, to the extent that manual control is present in many animals whose evolutionary past does not yet equip them with the capacity for speech, the capacity for co-manipulation may set the stage for co-articulation.

### Naturalistic observation

Granted that co-manipulation would be interesting to discover, how could one look for it? A first thought is to observe the microscopic features of manual behavior in the laboratory, taking advantage of technical systems for recording and quantifying properties of limb movements (e.g., Cai and Aggarwal, 1999). We have used such systems in our research (e.g., Studenka et al., 2012). However, the method we have generally favored has been simpler. We have preferred to observe behavior in situations where there are two easily observed ways of grasping any given object, especially when one of those ways can be plausibly linked to what will be done with the object. We like this approach because it can be pursued in the everyday environment, permitting or, better yet, *encouraging*, naturalistic observation.

It was, in fact, a naturalistic observation that paved the way for most of the research to be described in this article. While the first author was eating at a restaurant, he observed a waiter filling glasses with water. Each glass was inverted and the waiter had to turn each glass over to pour water into it. The waiter grasped each glass with his thumb *down*, whereupon he turned the glass over and poured water into the glass, holding the glass with his thumb *up*. Finally, he set the filled glass down, keeping his thumb up, and then proceeded to the next glass, turning his hand to the thumb-down position as he prepared for the next episode of glass filling.

The usual way of reaching for a glass is, of course, to take hold of it with a thumb-up posture. Why, then, did the waiter grasp the glass with his thumb down? Grasping an inverted glass with a thumb-down posture afforded a thumb-up hold when the waiter poured water into the glass and then set it down on the table. If the glass had been picked up with the thumb up, the resulting thumb-down posture would have made the subsequent pouring and placement awkward. At extreme forearm rotation angles (e.g., thumb-down angles) as compared to less extreme forearm rotation angles (e.g., thumb-up angles), rated comfort is lower (Rosenbaum et al., 1990, 1992, 1993), muscular power is lower (Winters and Kleweno, 1993), joint configuration variance is higher (Solnik et al., 2013), and maximum oscillation rates, which are critical for quick error correction, are lower as well (Rosenbaum et al., 1996). For any of these reasons, it made sense for the waiter to grasp each inverted glass as he did.

### Two-alternative forced choice procedure

The waiter’s adoption of a thumb-down posture was consistent with the model shown in Figure 2. The waiter’s decision to grasp the glass thumb-down shows that he was aware of (or had learned) what he would do next with the glass, so his action selection reflected second-order (or possibly higher-order) planning.

The waiter’s maneuver was detected in a single naturalistic observation, so it was important to replicate the result in the laboratory. The laboratory method that was used relied on the two-alternative forced choice procedure. The two alternatives per trial were readily categorized actions, either of which was possible for the task at hand but only one of which was typically preferred (or expected to be preferred) over the other.

The logic of the approach was to find out how often one alternative was favored over the other depending on the nature of the choice difference. The approach proved useful, as indicated in the raft of studies that have used it (Rosenbaum et al., 2012). In the present article, we cover some of the major results of this work, including several findings that emerged after preparation of the review article just cited. Specifically, we review (1) findings that have been obtained about choice of grasp *orientation*, both in neurologically typical and neurologically atypical adults and children and in non-human primates; (2) findings concerning grasp *locations* along objects to be moved; and (3) findings concerning selection of actions that involve walking as well as reaching.

### Grasp orientation in healthy young adults

**Figure 3:**
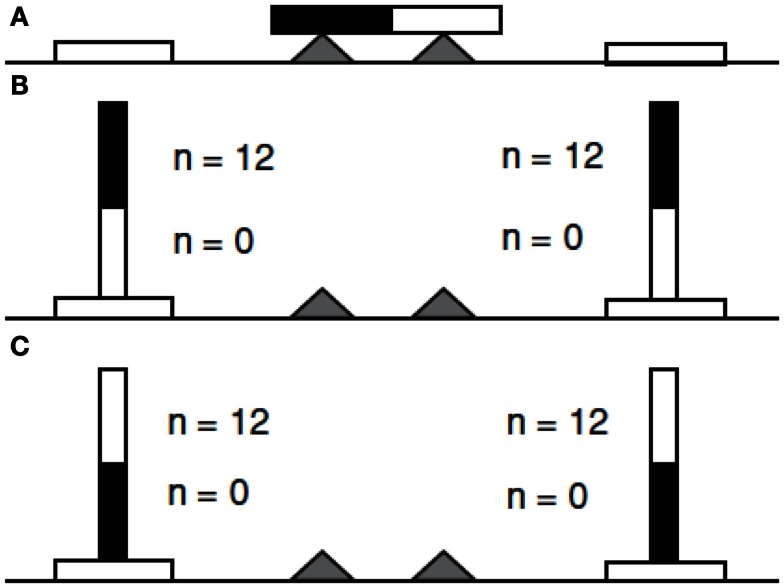
**Dowel task transport (Rosenbaum et al., 1990) demonstrating the end-state comfort effect**. In **(A)**, the black and white dowel rests on a cradle with a target on either side of the cradle. In **(B)** the dowel’s *black* end was to be placed in the left or right target. In **(C)**, the dowel’s *white* end was to be placed in the left or right target. The numbers near the black and white ends of the dowel represent the number of participants who grasped the dowel with the thumb directed toward that colored end of the dowel. (Image from Rosenbaum et al., 2006a.)

The first laboratory test of the tendency to select initially distinct grasp orientations in the service of later grasp orientations (Rosenbaum et al., 1990), involved presenting university students with a horizontally oriented wooden dowel resting on stands beneath the dowel’s ends (Figure 3). One end of the dowel was white; the other end was black. A circular disk target was placed on either side of the stand, closer to where the participant stood, and the participant was asked to reach out with the right hand to grasp the dowel and place either the black end or white end into a specified target. The task used a two-alternative forced choice method, though the two alternatives were not explicitly named for the participants. They could either grasp the dowel with an overhand (palm down) grasp, or they could grasp the dowel with an underhand (palm up) grasp. The dowel placement could likewise end in either of two ways: with a thumb-up posture or with a thumb-down posture. Ratings from the participants indicated that they found the thumb-down posture uncomfortable. In addition, they found the underhand (palm-up posture) less uncomfortable, and they found the overhand (palm-down posture) and thumb-up posture least uncomfortable (most comfortable).

For the action rather than the rating task, the main result was that participants consistently chose an initial grasp orientation that facilitated a thumb-up posture when the dowel was placed onto the target. When the participants were asked to place the black (left) end of dowel in the target, they picked up the dowel with an overhand grasp, which allowed them to end in a thumb-up orientation. By contrast, when participants were asked to place the white (right) end of the dowel in the target, they picked up the dowel using an *underhand* grasp, which also allowed them to end in the same thumb-up orientation. Regardless of the end that needed to be placed on the target, therefore, participants altered their initial grasps in a way that ensured a comfortable final grasp orientation. Rosenbaum et al. (1990) called this the *end-state comfort* effect.

**Figure 4:**
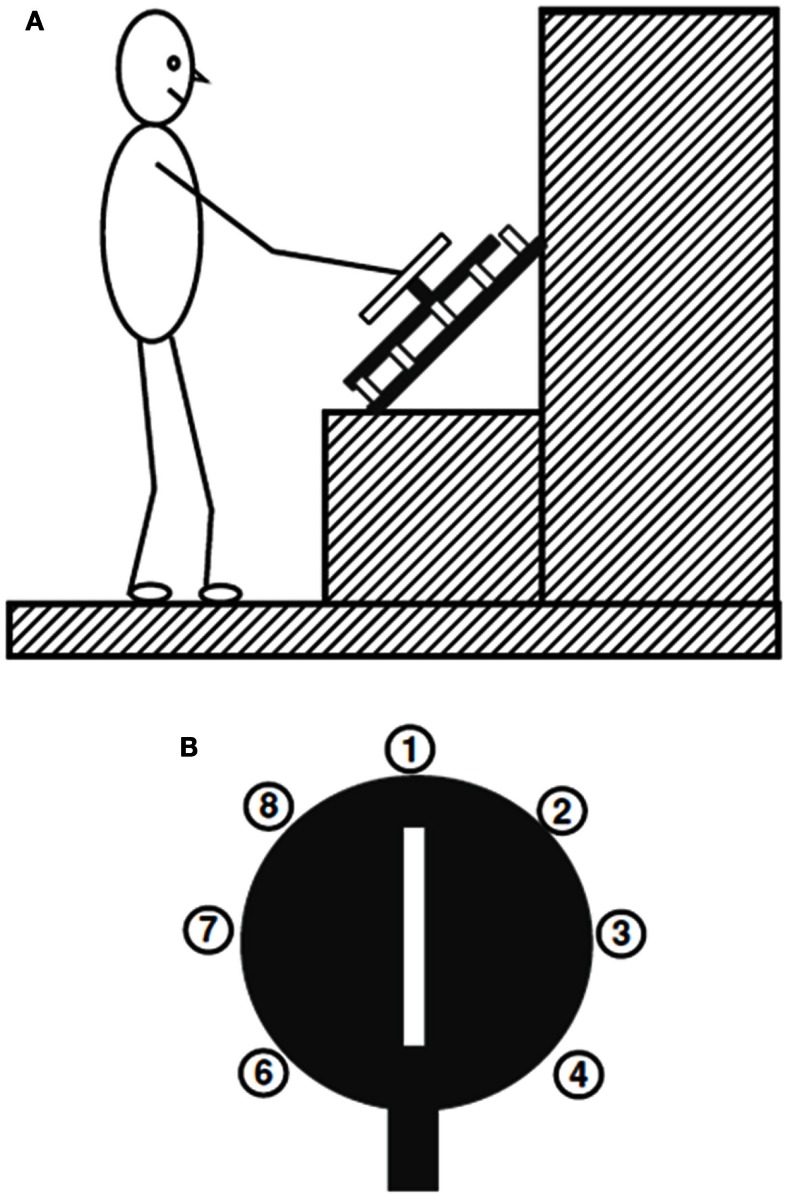
**Rotation task studied by Rosenbaum et al. (1993)**. In **(A)**, a participant stands in front of the wheel, which is oriented at a 45° angle, after reaching out and grasping the handle. In **(B)**, the numbered end locations for the tab (bottom) are depicted for all possible targets, except for 5, which is hidden by the tab. (Image fromRosenbaum et al., 2006a.)

After this first laboratory demonstration of the end-state comfort effect, many studies confirmed that the tendency to prioritize the grasp orientation at the end of a movement emerges in a wide variety of tasks. It was found that participants showed a preference for end-state comfort when the dowel task was reversed, so a vertical dowel was brought to a horizontal resting position; the final posture was a comfortable palm-down posture (Rosenbaum et al., 1990). When participants were asked to pick up an inverted cup and fill it with water, they chose an initially uncomfortable grasp and ended in a thumb-up grasp (Fischman, 1997). When participants were asked to grasp a handle and turn it to rotate a disk 180° so a tab would line up with a given location around the disk’s perimeter (see Figure 4), participants adopted initially uncomfortable grasps to ensure a comfortable grasp orientation at the end of the rotation (Rosenbaum et al., 1993).

The end-state comfort effect emerged not only in *single*-hand tasks, as just described, but also in *bimanual* tasks. In a bimanual version of the dowel transport task, participants grasped two horizontal dowels, one with each hand, and moved them to two vertical positions (Weigelt et al., 2006). Participants grasped the dowels in a way that afforded comfortable thumb-up grasps at the ends of the dowel transports. Participants in other experiments behaved similarly (Janssen et al., 2009, 2010).

Subsequent studies showed that precision rather than end-state comfort *per se* may be the decisive factor in second-order grasp planning. Short and Cauraugh (1999) showed that participants were less likely to grasp a dowel in a way that ensured end-state comfort if the target to which the dowel was moved was wide rather than narrow. A similar effect emerged in a unimanual disk rotation task study in which participants were asked to take hold of a handle in order to turn a lazy susan to an ending orientation (Rosenbaum et al., 1996). In one condition, securing the ending position took very little control, thanks to a bolt that stopped the disk’s rotation. In that condition, only half the participants showed the end-state comfort effect. By contrast, a much larger proportion of participants showed the end-state comfort effect when they had to control the final orientation through normal aiming.

All the results summarized in the last paragraph indicate that the term “end-state comfort” may be a misnomer. Ending in a comfortable state may be less important than occupying postures affording the most control. For further evidence, see Künzell et al. (in press).

### Grasp planning in non-human animals

The evidence just reviewed suggests that consideration of grasp orientation is an important constraint guiding action selection in object manipulation, at least in neuro-typical college students. Is this factor also important in other populations?

**Figure 5:**
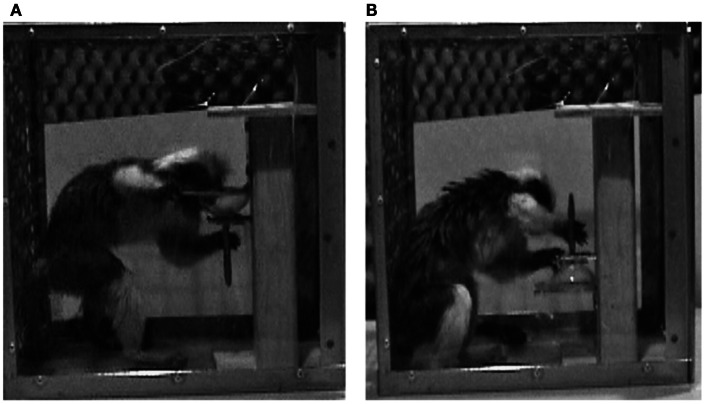
**A cotton-top tamarin performing the cub extraction task of Weiss et al. (2007)**. In **(A)**, the monkey grasps an upright cup’s stem using a canonical thumb-up posture. In **(B)**, the same monkey grasps the inverted cup’s stem using a non-canonical thumb-down posture. (Image from Weiss et al., 2007.)

Consider first performance by non-human primates. Studies of object manipulation in non-human primates have shed light on the evolutionary history of the cognitive capacities underlying manual action selection. Weiss et al. (2007) tested cotton-top tamarin monkeys on a modified version of the tasks described above. As shown in Figure 5, these cotton-top tamarins were presented with a food-baited champagne glass oriented upright or inverted. The base of the glass was removed and a long rod extended the glass’s stem. Both when the glass was upright or inverted, a flat plate prevented the monkeys from reaching into the glass to remove a marshmallow visible inside it. To retrieve the food, each individually tested monkey had to slide the glass toward him or herself to remove the marshmallow.

**Figure 6:**
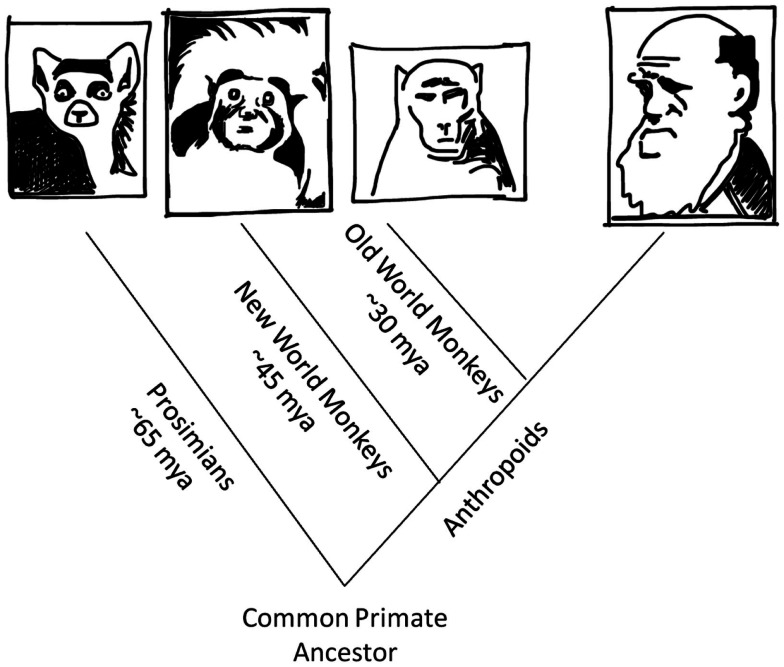
**Evolutionary tree stemming for a common primate ancestor to prosimians (e.g., the ring-tailed lemur shown here), which departed from the anthropoid line approximately 65 million years ago; to New World monkeys (e.g., the cotton-top tamarin shown here), which departed from the anthropoid line approximately 45 million years ago; to Old World monkeys (e.g., the macaque shown here), which departed from the anthropoid line approximately 30 million years ago; and to *Homo sapiens* (e.g., Charles Darwin shown here)**.

When the cup was upright, the tamarins grasped the stem with a typical thumb-up orientation. More interestingly, when the cup was inverted, the tamarins grasped the stem with an atypical thumb-down orientation. In the latter case (as in the former) the monkeys ended with the glass held thumb-up (see Figure 5). Thus, the tamarins, like college students and waiters, prioritized comfort (or presumed comfort) of final grasp orientations over prioritized comfort (or presumed comfort) of initial grasp orientations. This outcome suggests that the cognitive substrates for second-order grasp planning may have been in place as long as 45 million years ago, when the evolutionary line leading to tamarins diverged from the evolutionary line leading to humans (Figure 6).

**Figure 7:**
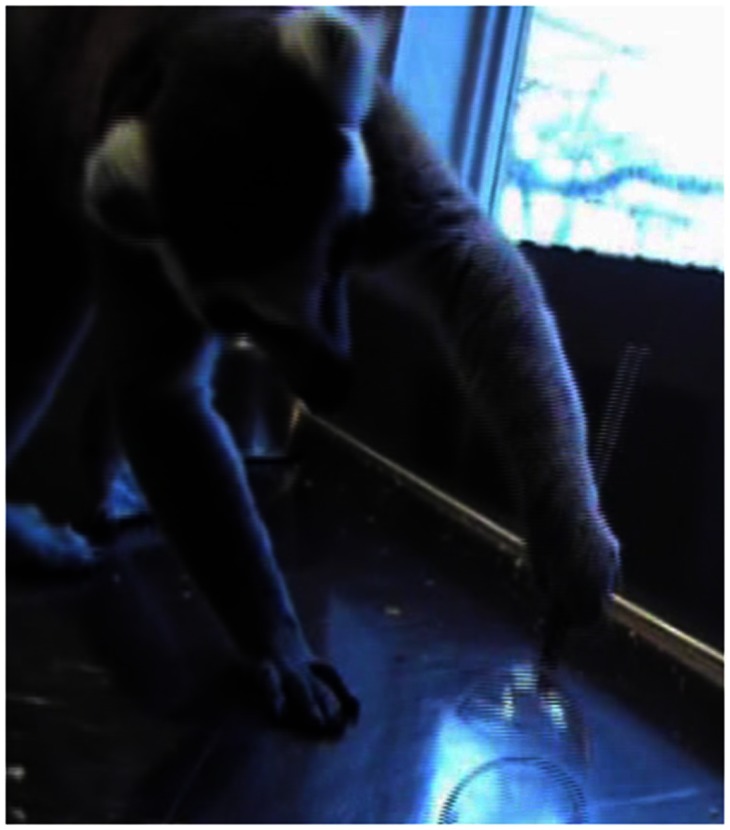
**A ring-tailed lemur grasping an inverted cup’s stem using a thumb-down posture**. The lemur then inverted the cup to remove a raisin from it. (Image fromChapman et al., 2010.)

Can the lineage for such planning be placed even farther back in time? Chapman et al. (2010) showed that it could. These authors obtained the same grasp-planning effect when the cup task (slightly modified) was used with lemurs. Lemurs are the most evolutionarily distant living primate relatives of humans (Figures 6 and 7). The lemur line diverged from the anthropoid line (the line leading to *Homo sapiens*) approximately 65 million years ago, or 20 million years earlier than for tamarins.

A final remark about evolution is that one would expect the planning ability indexed by grasp planning also to exist for old world monkeys and apes; otherwise, there would be a disconcerting “hole” in the picture. Rhesus monkeys (Nelson et al., 2011) and chimpanzees (Frey and Povinelli, 2012) also show sensitivity to future grasp orientation requirements, so as far as we can tell, then, the capacity for second-order grasp planning was in place as long as 65 million years ago and has held fast since that time.

### Grasp planning in babies, toddlers, and children

What about ontogenetic rather than phylogenetic development? In humans, the species whose ontogenetic development is of most interest to us, first-order grasp planning takes hold within the first year of life. Babies modify their grasps according to the properties of objects they reach for. The relevant literature is briefly reviewed in a textbook about motor control written mainly for psychologists (Rosenbaum, 2010) and at greater length in a recent handbook chapter (Savelsbergh et al., 2013).

In terms of the development of second-order grasp planning, such planning appears in some toddlers at around 18 months of age (Thibaut and Toussaint, 2010). Surprisingly, though, second-order grasp planning as studied in the manner outlined earlier does not reach adult-like competency until 9 or 10 years of age (Hughes, 1996; Smyth and Mason, 1997; Manoel and Moreira, 2005; Thibaut and Toussaint, 2010; Weigelt and Schack, 2010; Jovanovic and Schwarzer, 2011).

Several studies have also investigated child clinical populations. Autistic children and mildly learning-disabled children show less sensitivity to final grasp orientation than do age-matched controls (Hughes, 1996). Less consistent sensitivity to final grasping posture is also seen in children with cerebral palsy (Crajé et al., 2010) and in children with Williams’ syndrome (Newman, 2001).

### Grasp planning in adult clinical populations

Studies of adult clinical populations have also revealed grasp-planning deficits. In a task requiring participants to grasp a dowel and rotate it to different positions, individuals with visual agnosia did not consistently choose initial grasps that facilitated comfortable grasp orientations at the ends of the rotations (Dijkerman et al., 2009). Neither did adults with cerebral palsy (Crajé et al., 2009) or with apraxia due to unilateral lesions (Hermsdörfer et al., 1999). On a more up-beat note, adults with autism spectrum disorder exhibited some sensitivity to end-state comfort, not only in themselves but also in others, as shown in a study of handing a tool to another person (Gonzalez et al., 2013). The capacity for anticipating the needs of others was less consistent in the autistic individuals than in their typically developed age-matched peers, however.

### Grasp height

Constraints that come into play in planning for object manipulation are not only revealed by grasp *orientations*; they are also revealed by grasp *locations*. For example, when grasping a glass to place it on a high shelf, a person might grasp the glass low, near the base, to avoid an extreme stretch during the placement. Similarly, when grasping a glass to place it on a low shelf, the same person might grasp the same glass higher to avoid an extreme downward stretch.

These expectation were borne out in a naturalistic observation made by the first author at his home in the midst of returning a toilet plunger to its normal position on the floor. The details of the incident are unimportant. We will spare you! Suffice it to say that the type of manipulandum for which the phenomenon first appeared proved useful in laboratory experiments, where a fresh plunger was used.

**Figure 8:**
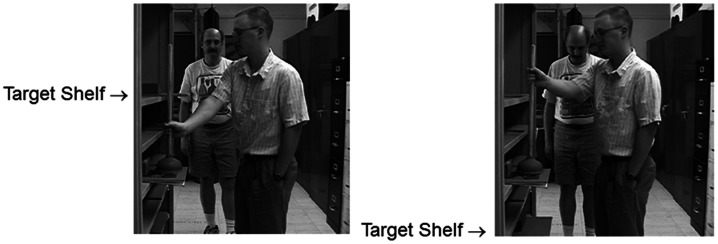
**Two of the conditions studied by Cohen and Rosenbaum (2004) in their demonstration of the grasp height effect**. The plunger occupies the same starting position in both conditions shown here (and in all five target-height conditions tested). Each participant was instructed to keep his or her left hand in his or her left pocket and to begin each trial with the right-hand hanging by the participant’s side. *Left panel*: the highest target shelf tested. *Right panel*: the lowest target shelf tested. The experimenter is the first author of the present article. The participant gave permission to have his face shown.

**Figure 9:**
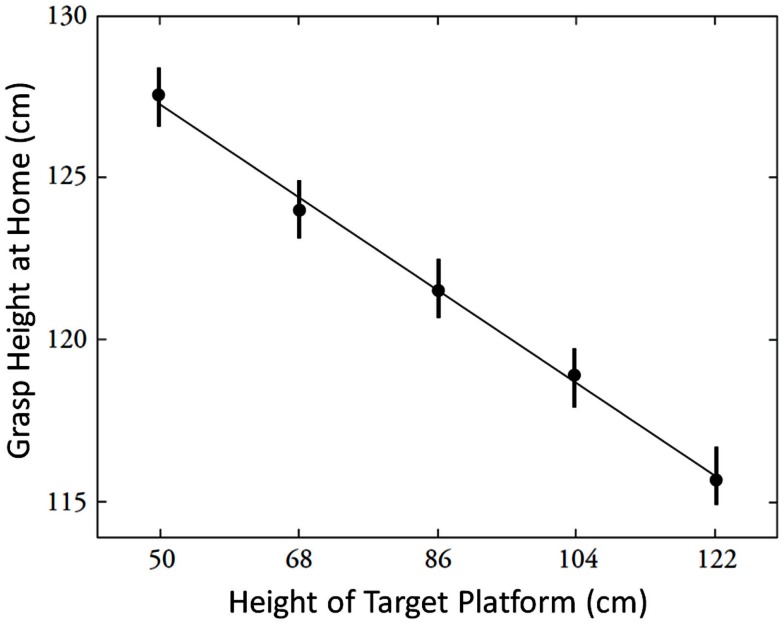
**The grasp height effect**. FromCohen and Rosenbaum (2004).

Participants were asked to place the plunger onto shelves of different heights (Cohen and Rosenbaum, 2004). As seen in Figure 8, the plunger always began at the same location. The participant was asked to take hold of it with the right hand in order to move it to another shelf of variable height. When the plunger was grasped to be placed on a high shelf, the grasp was low. Conversely, when the plunger was grasped to be placed on a low shelf, the grasp was high. In general, as seen in Figure 9, there was an inverse linear relation between target height (the independent variable) and grasp height (the dependent variable) within the range of home and target-heights studied.

This observed relation, which Cohen and Rosenbaum (2004) called the *grasp height* effect, can be understood to reflect a desire to avoid extreme joint angles, similar to what was seen for the hand orientation effects described earlier. Also as for the hand orientation effects, it turned out that required precision played an important role. The grasp height effect was attenuated when placement of the plunger on its target location required less precision than when placement of the plunger on its target location required a lot of precision (Rosenbaum et al., 2006a). This outcome suggested that avoidance of extreme joint angles was sought when greater control was needed, as concluded earlier in connection with grasp orientations.

Another finding from the study of Cohen and Rosenbaum (2004) shed light on the nature of the action selection process. Cohen and Rosenbaum found that grasp heights depended not just on upcoming task demands but also on previous actions. After the plunger was brought from its home position to the target, the participant lowered his or her hand and then returned the hand to the plunger to bring the plunger back to the home position. When participants did this, the grasp heights they adopted were very similar to the grasp height just adopted for the home-to-target grasps (Cohen and Rosenbaum, 2004). Thus, participants did not strive for invariant end postures for the target-back-to-home transports. If they had, they would have grasped the plunger higher from high targets than they originally did (overcoming the tendency to grasp low for high targets at the home site), and they would have grasped the plunger lower from low targets than they originally did (overcoming the tendency to grasp high for low targets at the home site). What participants did instead was to grasp close to where they had just grasped the plunger when they brought it to the target from the home position.

A subsequent study showed that it was the location on the plunger shift rather than the posture that participants recalled for the return moves. Weigelt et al. (2007) showed this by having participants step up onto, or down from, a stool after moving the plunger from the home to the target and before returning to the home position. Instead of adopting a posture like the one adopted when holding the plunger on the target (just before releasing it), which would have meant holding the plunger at a different point along the shaft after stepping up or down, participants grasped the plunger close to where they had grasped it before, even if this required a very different posture. Thus, participants relied on memory of the grasp location to guide their grasps for the return trip. Relying on that strategy may have required fewer cognitive resources than planning a new action from scratch every time. How different a posture would be tolerated for the return trip is still an open question.

## Reaching and Walking

The studies reviewed above concerned choices of grasps for forthcoming object manipulations. The studies provided evidence for second-order planning at least. The studies showed that grasps are not just adjusted according to the immediate demands of taking hold of an object based on its currently perceived properties (first-order planning), but instead also depend on what will be done with the object afterward. For evidence that grasp planning can go beyond the second-order, see Haggard (1998).

Grasp features are not the only aspects of behavior that provide evidence for higher-order object-manipulation planning. Consider a study by Studenka et al. (2012). They asked participants to engage in the everyday task of opening a drawer to grasp an object inside. When participants knew that no object had to be grasped (i.e., they would simply open the drawer) they held the grasping arm lower for the drawer opening than when they knew they would lift an object from the drawer after opening it. Not only was the arm higher in the lifting condition than in the non-lifting condition; the joint angles were also more similar to those that would be adopted for the lift. This outcome is similar to the grasp height effect of Cohen and Rosenbaum (2004) in that it reflects assimilation: features of upcoming behavior are reflected in behavior that comes before. Such assimilation reflects the tendency to minimize differences between immediately forthcoming postures and subsequent postures, as shown in Figure 2.

### Standing for object manipulation

Whereas Studenka et al. (2012) looked at arm configurations, it is also possible to look at more macroscopic aspects of behavior to draw inferences about action planning in the context of object manipulation. Specifically, it is possible to study how people approach a space where they know they will manipulate an object. People approaching the space must project themselves to a new position, often in a very different part of space than the one they currently occupy. How they do so is a topic of longstanding interest in psychology.

Little research has been done on whole-body planning of object manipulation, but some work has been done on it in our lab at Penn State. van der Wel and Rosenbaum (2007) asked how people walk up to a table to move a plunger to the left or right over a long or short distance. The long distance was 120% of the subject’s arm length; the short distance was 20% of the subject’s arm length. Participants began each trial standing some distance from the table: one, two, three, or four steps from the table, where “steps” were defined for each participant based on his or her height. Participants could use whichever hand they wished to perform the plunger displacement task, which involved lifting the plunger and then setting it down the long or short distance away toward the left or right.

The main result was that participants preferred to stand on the foot opposite the direction of forthcoming object displacement if the displacement was large. If the displacement was small, participants displayed no foot preference at the time of manual displacement.

Why did participants stand on the opposite foot for large displacements? Doing so made it possible for participants to rock in the direction of the upcoming manual displacement, landing on the foot ipsilateral to the placement. No such rocking was observed when the manual displacements were small.

**Figure 10:**
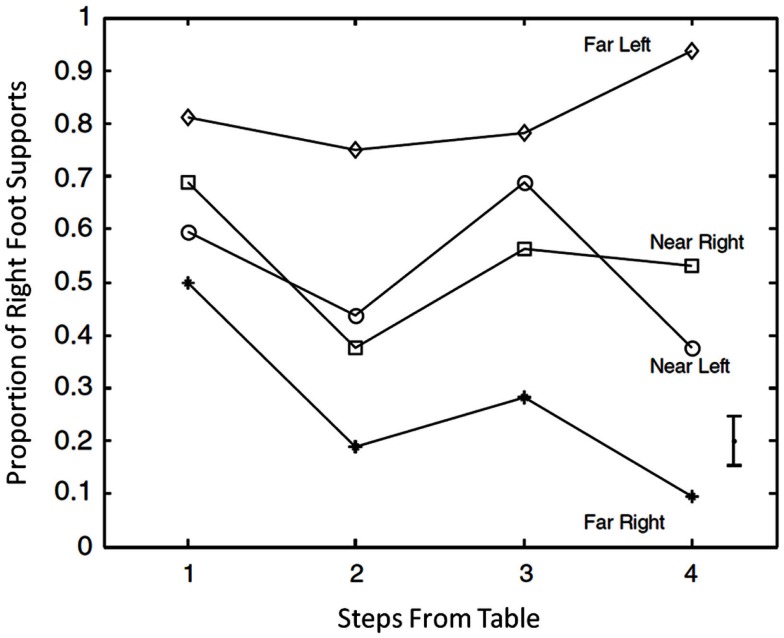
**Mean observed proportion of trials in which participants stood on the right foot when they grasped a plunger to move it far to the left, near to the left, near to the right, or far from the right, plotted as a function of the distance from the table at the start of each trial**. Fromvan der Wel and Rosenbaum (2007).

What was the effect of the participants’ initial distance from the table? To the surprise of van der Wel and Rosenbaum (2007), there was a stronger preference to stand on the foot contralateral to the large forthcoming object shift when participants initially stood *far* from the table than when participants initially *near* the table (Figure 10). The reason for this outcome was not entirely clear. Participants may have thought they could not navigate as well to position their feet as they wished when they began close to the table. With more steps, however, they may have had had more of a chance to adjust their foot positions.

The latter hypothesis was confirmed through an analysis of changes in step lengths as a function of starting distance from the table. van der Wel and Rosenbaum (2007) found that the greater the starting distance, the more the step lengths changed as participants approached the table. So as participants approached the table, they altered their steps to afford contralateral foot support at the time of the large manual displacement.

These results, along with the others summarized in this section, suggest that participants could project themselves into the positions they would need (or want) to adopt for the manual transfers they would perform.

### Walking for object manipulation

If people can mentally project themselves *to* future body positions, might they also be able to project themselves moving *through* those positions? Might they, in other words, be able to imagine themselves carrying out object manipulations while moving through the environment – for example, while grabbing items from a supermarket shelf during a trip down the aisle?

**Figure 11:**
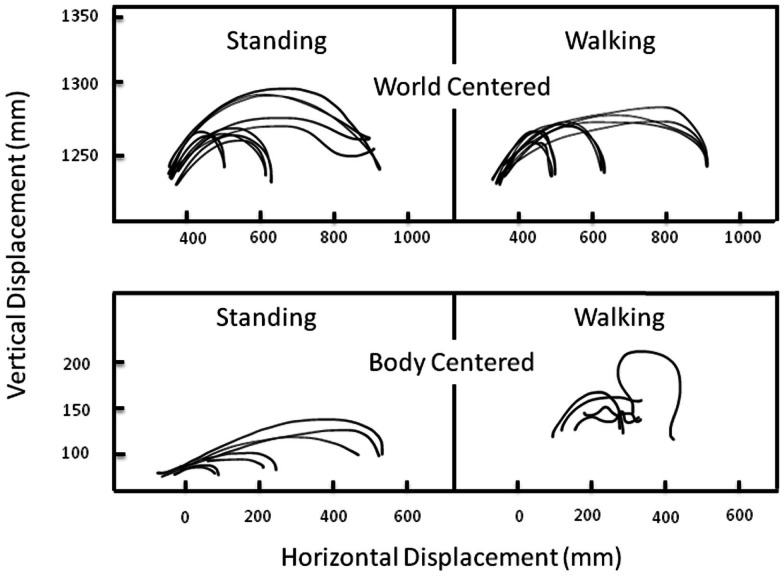
**Vertical displacement of a hand-held cup as a function of horizontal displacement of the same cup when standing (left column) or walking (right column) and when the data are plotted in extrinsic spatial coordinates (top row) or intrinsic joint coordinates (bottom row)**. Adapted fromMarteniuk and Bertram (2001).

That people can coordinate their reaching and walking in cognitively impressive ways was shown by Marteniuk and Bertram (2001), who compared hand trajectories produced by people moving a cup from one position to another either while standing still or while walking. As seen in Figure 11, the hand paths were virtually identical in the two cases, at least when the hand paths were depicted in external, spatial coordinated. When the hand paths were depicted in intrinsic, joint-based coordinates, they were strikingly different.

This finding is reminiscent of a classic result reported 20 years earlier. In that study, Morasso (1981) found that hand paths for point-to-point reaching movements were nearly straight in extrinsic spatial coordinates but were often curved and highly complex in intrinsic, joint-based coordinates. Morasso’s result suggested that the motor system puts a premium on generating movements defined with respect to external coordinates. The complexity of motions in intrinsic coordinates suggests that the intrinsic control system – the one responsible for moving and stabilizing muscles – is extremely “clever,” somewhat like a highly skilled secretary who works behind the scenes to keep his or her boss looking good (Rosenbaum and Dawson, 2004). In the case of Marteniuk and Bertram’s (2001)result, the fact that the motor system could generate simple hand paths in extrinsic space even when people were *walking* is a stunning result. Developing a computational model capable of simulating this capability will be a worthwhile aim for future research.

In the walk-and-reach study of Marteniuk and Bertram (2001), the topic of interest was participants’ ongoing behavior. An issue that was not addressed in that report was how far in advance people planned their walks and reaches. That is a topic for which most of the research we know of has come from our own laboratory, where again we have found it useful to rely on the two-alternative forced choice procedure.

**Figure 12:**
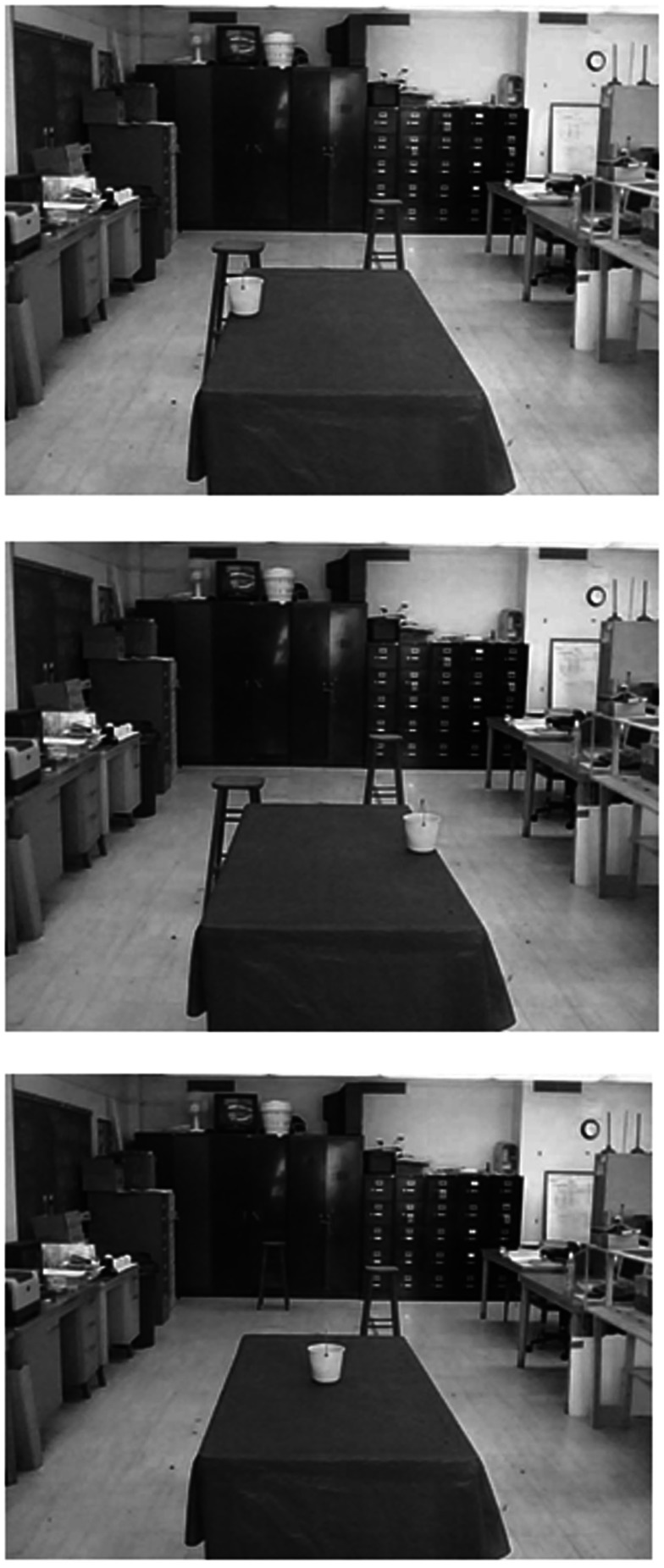
**Three arrangements used by Rosenbaum et al. (2011) to study walking and reaching**. In all cases, the participant stood at the site where these photographs were taken. *Top panel*: bucket near the left edge of the table and the left target stool is nearby. *Middle panel*: bucket near the right edge of the table and the left target stool is again nearby. *Bottom panel*: bucket in the middle of the table and the right target stool is nearby. Adapted fromRosenbaum (2012).

In one of our experiments (Rosenbaum et al., 2011), we asked participants to pick up a child’s beach bucket on a table and carry it to either of two sites beyond the table (Figure 12). To pick up the bucket, the participant could either walk along the left or right side of the table. If the participant walked along the left side of the table, he or she was supposed to pick up the bucket with the right hand and carry it to a target site (a stool) beyond the table’s left end. If the participant walked along the right side of the table, he or she was supposed to pick up the bucket with the left hand and carry it to a target site (a different stool) beyond the table’s right end. In different trials, the left and right target sites (the left and right stools) occupied different distances from the end of the table. Crossed with this variable, the bucket was close to the left edge of the table, in the middle of the table, or close to the right edge of the table.

Given these possible arrangements, it was possible to study the costs of walking versus reaching. In some conditions, participants had no conflict between these two costs. For example, participants had no conflict if the bucket was near the left edge of the table, the left target was close, and the right target was far away (top panel of Figure 12). However, if the bucket was near the right edge of the table, the left target was nearby, and the right target was far away (middle panel of Figure 12), participants had a conflict. In that case, participants could either walk along the right side of the table, reaching less but walking more, or they could walk along the left side of the table, reaching more but walking less. Finally, in terms of the examples reviewed here (just some of the conditions tested), if the bucket was in the middle of the table and the left target was farther away than the right (bottom panel of Figure 12), participants could walk less by walking along the right side of the table, or they could walk more, walking along the left side of the table, reaching just as far in both cases. If they walked more, they would have to use the less favored hand (the left hand for the participants in this study).

**Figure 13:**
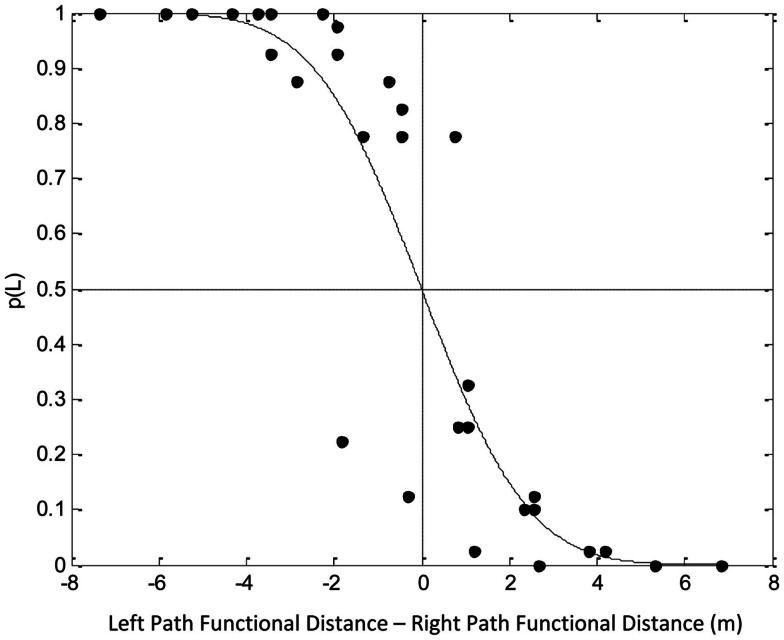
**Probability, *p*(*L*), of choosing to walk along the left side of the table (Rosenbaum, 2012). Left path functional distance was defined as walking distance + 10.3 × right-hand reaching distance**. Right path functional distance was defined as walking distance + 12.3 × left-hand reaching distance, all in meters (m). Adapted fromRosenbaum (2012).

So what was more important, walking less or reaching with the hand that was preferred? With the tasks used, which go beyond those reviewed above, Rosenbaum et al. (2011) could estimate the relative costs of walking over some distance versus reaching over some distance, and they could estimate the relative cost of reaching with the left hand or right. The way they estimated the relative costs is reflected in Figure 13, which shows the probability, *p*(*L*), that participants walked along the left side of the table plotted as a function of the difference between two derived measures, “left path functional distance” and “right path functional distance.” Left path functional distance was defined as the sum of the walking distance (in meters) plus the right-hand reaching distance (also in meters), with the latter term being multiplied by an empirically fit constant. Similarly, right path functional distance was defined as the sum of the walking distance (in meters) plus the left-hand reaching distance (also in meters), with the latter term being multiplied by another empirically fit constant. The empirically fit constant for the right hand was 10.3. The empirically fit constant for the left hand was 12.3. Based on these two values, it was possible to say that right-hand reaching was less costly than left-hand reaching (10.3 compared to 12.3) and that reaching over some distance was much more costly than walking over that same distance, 11.3 times more costly, in fact (the mean of 10.3 and 12.3).

Two further remarks are worth making about the study just reviewed. First, the study was aimed at showing how different kinds of costs are considered together. *A priori*, it is not obvious how walking costs and reaching costs are co-evaluated in the planning of walking and reaching. The study just summarized shows that it is possible to find a common currency for evaluation of the two kinds of costs. That common currency is (or is analogous to) “functional distance,” defined as the weighted combination of walking distance and reaching distance. Presumably, the weights would change if walking were challenged more (e.g., by adding leg loads) or if reaching were challenged more (e.g., by adding wrist loads). Being able to estimate mathematical weights such as these is central to the general approach outlined here because, as stated in the introduction of this paper, we believe that tasks can be represented as vectors of weights for dimensions on which tasks vary (see Figure 2).

The second remark is that the study just reviewed was done by having participants actually walk and reach in the environment depicted in Figure 12. The study was later repeated by showing a new group of participants (another group of Penn State undergraduates) pictures of the environment in which the real task had been done, photographed from the perspective of someone standing where participants stood at the start of each real-action trial (Rosenbaum, 2012). Examples of those images are shown in Figure 12. In each experimental trial, one image was shown on a computer and the participant either pressed a left key, indicating that s/he would walk along the left edge of the table (carrying the bucket with the right hand to the left target), or the right key, indicating that s/he would walk along the right edge of the table (carrying the bucket with the left hand to the right target). There was no time pressure, just as in the “real-action” experiment. Moreover, participants were allowed to hold and heft the bucket (which was empty) before doing the computerized “virtual-action” task.

The result of the virtual-action study was that the choices participants made when they indicated how they *would* do the task were almost identical to the choices made by participants who actually *did* the task. The result lent credence to the impression that Rosenbaum et al. (2011) had when they ran their experiment, that their real-action participants knew which way they would go as soon as they left the start point.

It also happened that in the virtual-action study of Rosenbaum (2012), the choice reaction times were longer the more similar the functional path lengths of the left and right paths. This outcome let Rosenbaum (2012) reject the hypothesis that participants mentally simulated one task alternative and then the other, choosing whichever seemed easier. Such a serial simulation method would have resulted in a different pattern of choice reaction times than the one obtained. The choice reaction times would have grown with the sum of the left and right functional path lengths rather being inversely related to the difference between the two path lengths, as found.

Did it make sense that participants did not rely on serial simulations to choose their actions in this reach-and-walk task? Rosenbaum (2012) suggested that it did. By analogy to someone being chased by a tiger, if you had a tiger on your tail, the tiger would have you for lunch if you fully simulated alternative escape paths. If you stood at a choice point, blithely imaging yourself going one way or the other, you would probably land in the tiger’s jaws. A more efficient method would be to compare critical differences between the paths, quickly choosing your action based on differences between the alternatives. The time to choose between the paths would grow with their similarity, as was found in the virtual-action task of Rosenbaum (2012) and as is typically found in studies of perceptual discrimination (e.g., Johnson, 1939).

## Conclusion

The research summarized in this article has been concerned with choosing between actions expressed at the relatively low level of carrying out movements, especially with the hands and legs in the context of object manipulation. As noted in the introduction, there has been relatively little attention paid to the motor system in psychology, which is odd considering that psychology is the science of mental life and behavior, whereas motor control is the science of how one gets from mental life *to* behavior.

The latter definition might not be the one that most motor-control researchers spontaneously provide when asked to define their field, for most motor-control researchers typically come from engineering or neuroscience. That issue aside, it is not always clear that to understand motor control, one must invoke mental states. Some aspects of motor control are explicitly removed from mental states in that they rely on mechanical properties of the neuro-muscular and skeletal system, sometimes obviating the need for planning or control, as discussed in Section “Mechanics.” Similarly, reflexive (highly automatic) responses might not require extensive mental involvement. Even in the case of simple tasks where reflexes seem sufficient, mental states turn out to have a tuning function, as reviewed in Section “Coupling.” Thus, mental states are essential for motor control, just as motor control is essential for the expression of mental states.

Why motor control has received short shrift in psychology is an interesting topic that, among other things, tells psychologists about their values (Rosenbaum, 2005). One hypothesis about why psychologists have not pursued motor-control research as actively as they might have is that they think the methods that are needed are extremely technical, so that, to make any kind of progress, one has to record the electrical activity of muscles, for example, or the detailed kinematic properties of the limbs with expensive equipment. As we have tried to show here, however, simple behavioral methods can be profitably applied to the study of motorically expressed action choices. None of the studies described here (from our lab) required exotic or highly technical equipment. The equipment that was needed to do the studies we have summarized has been limited to tables, stools, beach buckets, toilet plungers, wooden dowels, wooden disks, webcams, and laptop computers.

Even with such primitive materials, however, we have arrived at some useful conclusions. The first of these is that different tasks can be represented in terms of the weights assigned to different performance variables. No matter how obvious this point is, it actually diverges from a prevailing view in engineering-inspired motor-control research – namely, that there is some single optimization variable that governs motor control. Various candidates for this single optimization variable have been suggested over the years, including minimization of mean squared jerk (Hogan, 1984), minimization of mean squared torque change (Uno et al., 1989), and minimization of endpoint variance (Harris and Wolpert, 1998). But movements do not always satisfy these constraints. Indeed, the flexibility of performance – for example, the possibility of making high-jerk bow strokes while playing the violin with staccato style versus making very smooth bow strokes while playing the violin with legato style – reflects the opposite of unstinting loyalty to one fixed optimization constraint. Rather, it reflects the possibility of re-prioritizing constraints according to the task to be achieved. The essence of skill, we believe, is being able to re-prioritize constraints, not being locked into prioritizing constraints in a fixed fashion.

Our second conclusion is that identifying the priorities of constraints for a task need not be viewed as an elusive goal. Instead, it is a reachable goal if one is willing simply to try to find out which means of achieving a task are preferred over others. All the presently reviewed experiments (from our lab) had this goal. What was common to all the experiments was the aim of determining which performance variables participants cared about more than others. To answer this question, we relied on ratings, measures of performance quality, and, especially, two-alternative forced choice preferences.

Our third and final conclusion concerns embodied cognition. This has become a very popular topic lately. The embodiment perspective is one that we find congenial given our interest in motor control, but the discussion of embodiment has glossed over the details of motor performance. Saying that perception implicitly calls up a response is fine as far as it goes, but a “response” is actually an equivalence class of possible movement solutions, as detailed here. Therefore, turning to a familiar example from the embodied-cognition literature (Glenberg and Kaschak, 2002), reading a sentence about opening a drawer may evoke a drawer-opening response, but there isn’t a single movement that achieves drawer opening, as discussed earlier in connection with the study of Studenka et al. (2012). Likewise, saying that embodiment may entail simulating actions is only theoretically helpful up to a point. As we have argued here in connection with the study of choosing between walking-and-reaching routes (Rosenbaum, 2012), simulation may not be used, at least judging from the fact that the time to choose between the routes was not predicted by the sum of their lengths. Other studies from our lab, not reviewed here (Walsh and Rosenbaum, 2009; Coelho et al., 2012) have also cast doubt on a naïve account of motor imagery according to which actions are chosen by running mental movies of the actions in order to find out which is better or best; see also Cisek (2012). Were such a method to be used, we probably would not have survived in the jungles from which we evolved. The methods we use to choose actions are honed by eons of selective pressure. The features of actions that are preferred are ones that have been selected for and that the experiments summarized here have been aimed at identifying.

## Conflict of Interest Statement

The authors declare that the research was conducted in the absence of any commercial or financial relationships that could be construed as a potential conflict of interest.
